# Dynamic Functional Connectivity of EEG: From Identifying Fingerprints to Gender Differences to a General Blueprint for the Brain's Functional Organization

**DOI:** 10.3389/fnins.2021.683633

**Published:** 2021-08-11

**Authors:** Ute Gschwandtner, Guy Bogaarts, Menorca Chaturvedi, Florian Hatz, Antonia Meyer, Peter Fuhr, Volker Roth

**Affiliations:** ^1^Department of Neurology and Neurophysiology, University Hospital of Basel, Basel, Switzerland; ^2^Department of Mathematics and Computer Science, University of Basel, Basel, Switzerland

**Keywords:** electroencephalography, dynamic functional connectivity, Parkinson disease, subject identification, gender classification analysis

## Abstract

An individual's brain functional organization is unique and can reliably be observed using modalities such as functional magnetic resonance imaging (fMRI). Here we demonstrate that a quantification of the dynamics of functional connectivity (FC) as measured using electroencephalography (EEG) offers an alternative means of observing an individual's brain functional organization. Using data from both healthy individuals as well as from patients with Parkinson's disease (PD) (*n* = 103 healthy individuals, *n* = 57 PD patients), we show that “dynamic FC” (DFC) profiles can be used to identify individuals in a large group. Furthermore, we show that DFC profiles predict gender and exhibit characteristics shared both among individuals as well as between both hemispheres. Furthermore, DFC profile characteristics are frequency band specific, indicating that they reflect distinct processes in the brain. Our empirically derived method of DFC demonstrates the potential of studying the dynamics of the functional organization of the brain using EEG.

## 1. Introduction

Due to advances in functional neuroimaging such as resting-state fMRI, there has been an increased focus on studying FC on an individual basis rather than at a group level (Dubois and Adolphs, 2016). It has recently been shown that FC profiles are highly individual, which emphasizes that it is indeed possible to draw inference on individual subjects (Finn et al., 2015). Similarly, the shape of an EEG spectrum qualifies as a statistical signature of a person (Näpflin et al., 2007). Another recent advance in the field of FC research is the recognition of information contained in the temporal dynamics of functional connectivity (Hutchison et al., 2013; Allen et al., 2014; Calhoun et al., 2014; Bassett and Sporns, 2017). This dynamic FC exhibits highly structured spatiotemporal states in which distinct patterns of FC recur across time and across subjects (Allen et al., 2014). These states are associated with cognition (Gonzalez-Castillo et al., 2015), consciousness (Barttfeld et al., 2015), neuropsychiatric disorders (Damaraju et al., 2014), personal traits (Shiino et al., 2017), and development (Hutchison and Morton, 2015). Furthermore, these FC states, when measured with fMRI, are associated with distinct EEG spectral signatures (Allen et al., 2018). As illustrated by other studies as well, EEG/MEG can be used to substantiate fMRI FC findings by providing an empirical link to a neural basis underlying BOLD signal changes (Tagliazucchi et al., 2012; Yu et al., 2016). Using a modeling approach, Honey and colleagues showed that the transient synchronization of neural populations is closely correlated with ultra slow fluctuations in the BOLD signal (Honey et al., 2007). Furthermore, they showed that when long time windows are used to calculate FC, the FC pattern is shaped by the underlying topology (Foster et al., 2016). Indeed, as pointed out by Allen and colleagues, the majority of EEG-fMRI integration studies have focused on EEG amplitude/FC modulations in relation to BOLD signal changes (de Pasquale et al., 2012; Chen et al., 2013; Liu et al., 2017; Allen et al., 2018). However, such a comparison is not straightforward because the relation between EEG power and actual spatial integration, as measured through spike-count correlations, is non-linear (Snyder et al., 2015). FC can also be studied using EEG/MEG exclusively and can be calculated over much shorter time-scales compared to fMRI (Van Diessen et al., 2015). This means that information about transient synchronization of neural populations, lasting between 100 and 300 ms (Varela et al., 2001), can be observed directly using EEG. Furthermore, using EEG, synchronization in different frequency bands can be analyzed separately, whereas in the BOLD signal these are lumped together. In this study we propose a novel EEG-based functional neuroimaging method to study the organization of transient functional connectivity. This method quantifies the correlation strength between time-varying FC signals, referred to as dynamic functional connectivity (DFC). Using EEG data from 105 healthy subjects scanned on two occasions up to 1 year apart, we show that, similar to an fMRI-derived FC profile (Finn et al., 2015), a DFC profile obtained from one session can be used to uniquely identify a given individual from a set of profiles obtained in a second session. Furthermore, we show that differences between male and female DFC profiles are substantial, which enables the prediction of a subject's gender based on his or her DFC profile. We also show that EEG-derived DFC profiles are organized according to a highly structured blueprint that is not only shared among people, but also across both hemispheres of the brain. Using additional data from a cohort of 81 Parkinson's disease (PD) patients, we show that our findings not only generalize to a state of health, but also of disease. Finally, our results also indicate that DFC could be used to distinguish PD patients from healthy controls.

## 2. Methods

### 2.1. Subject Information

The data set used in this study comprises three subsets from different studies conducted at the department of clinical neurophysiology, Basel University hospital and the Mathematics and Informatics department of Basel University. The first is from an ongoing SNFS study, investigating cognitive decline in Parkinson's disease (“Computer aided Methods for Diagnosis and Early Risk Assessment for Parkinson‘s Disease Dementia,” grant number 159682). Data from both healthy controls as well as PD patients was used. The other two subsets consist of data from the SNSF projects (“Improved prediction and monitoring of CNS disorders with advanced neurophysiological and genetic assessment,” grant numbers 124115 & 140338). All participants provided written informed consent in accordance with a protocol approved by the local ethics committee (Ethikkommission beider Basel). The 3 data sets used are described next. Data set 1. This data set consists of data from 33 healthy subjects (15 females, age 53–76) and 81 PD patients (28 females, age 45–84). EEG data were registered at baseline and again after 4 weeks and 6 months. At the time this study began, follow-up data from 25 healthy subjects and 57 PD patients was available. Baseline and 6 m data from the 33 healthy subjects and baseline and 4 w data from the 57 PD patients was used for the subject identification analysis. In case of the PD patients, we choses to use 4 w data instead of 6 m data because it is not yet known what influence a neurodegenerative disease such as PD has on a patient's DFC profile. Therefore, taking the shortest time-span between scan sessions minimizes the possibility of introducing bias.

#### 2.1.1. Data Set 2

EEG data from 40 healthy subjects (30 females, age 20–49) was registered on 3 occasions over a period of 2 years (baseline, 1y, 2y). Baseline and 1y data from all 40 subjects was used for the subject identification analysis.

#### 2.1.2. Data Set 3

EEG data from 41 healthy subjects (22 females, age 53–76) were registered at baseline and again after 3, 15, and 30 months. Baseline and 15m data were used for the subject identification analysis. For all 41 subjects a baseline scan was available. However, due to some dropouts, 15 m data were only available for 38 subjects. For the remaining 3 subjects 3 m data were used instead.

In all our analyses only data from healthy subjects were used. Data from the PD patients were used as an external validation set. For the analysis of differences between healthy subjects and PD patients, only data from sets 1 and 3 were used because the subjects from set 2 were significantly younger compared to the cohort of PD patients.

### 2.2. Data Acquisition and Preprocessing

**Figure 1:**
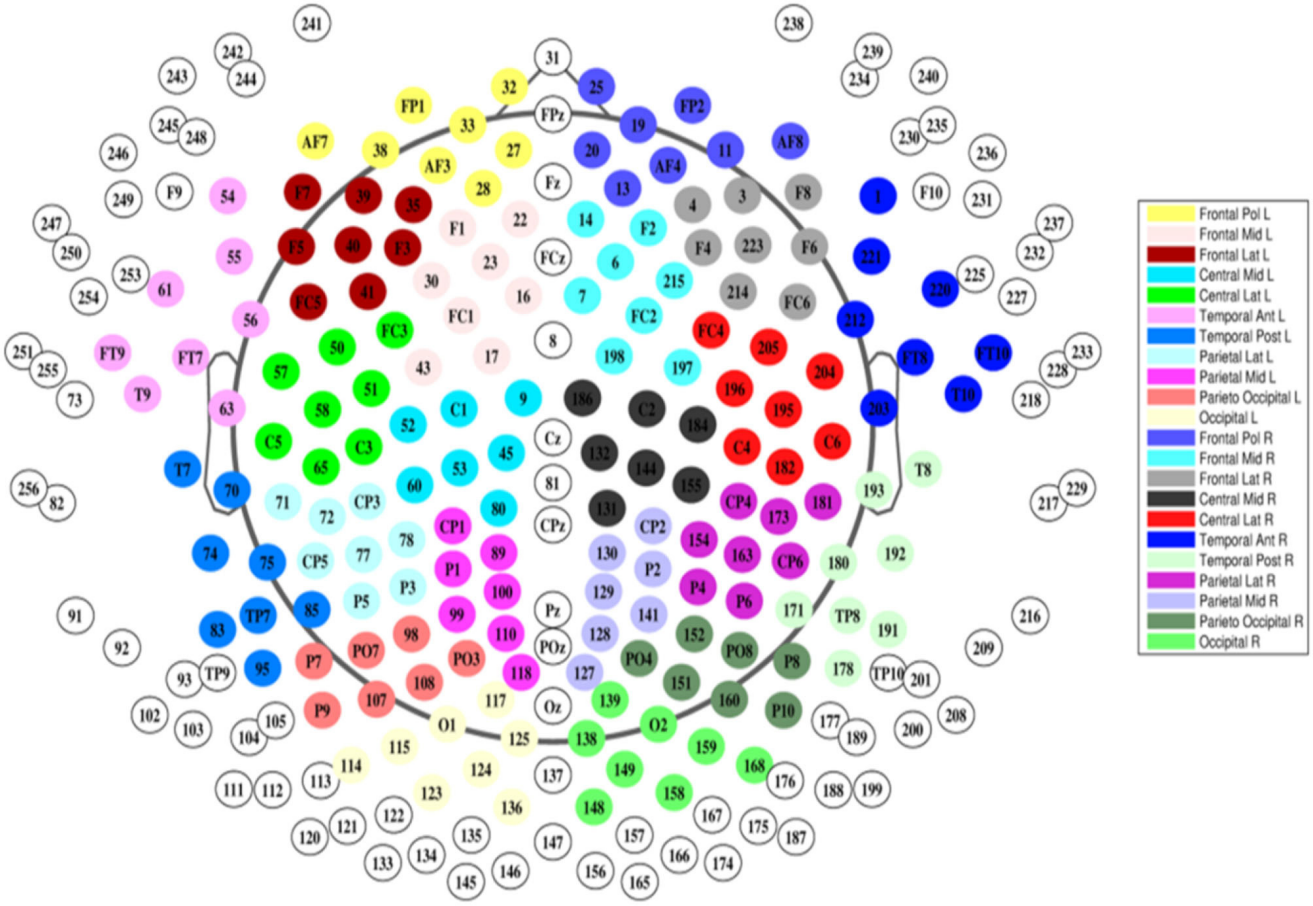
Our electrodes were grouped according to the topographical localization of the channels and then divided into 22 non-overlapping regions of interest corresponding to the frontal, central, temporal, parietal and occipital areas (bilaterally) (Hatz et al., 2017).

EEG was recorded using a 256-electrode Sensor Net^®^. (Geodesics). First, the correct Sensor Net size was determined by measuring the subject's head circumference. Next, the net was placed over the subject's head such that the central electrode (Cz) was located at the crossing of the midline and lateral line. Subjects were scanned sitting upright and were instructed to sit still. EEG data from all subjects was acquired using the same protocol, which consisted of 4 consecutive sessions. In the first sessions the subject is instructed with an audio cue to alternatingly open and close his or her eyes. This sessions is followed by 15 min of eyes-closed resting state, and subsequently 5 min of eyes-open resting state sessions, after which the first sessions is repeated once more. Total registration duration was around 30 min including instructions. Only data from the 15 minutes eyes-closed were used in this study. Raw EEG signals were recorded with a sampling rate of 1,000 Hz and filtered with a high-order, linear-phase, finite-impulse response filter (MATLAB: Firls, 0.5–70 and 50 Hz notch, filter order: 4.8 × sampling rate). Only 170 out of 256 electrodes were used, midline electrodes and electrodes located in the face and around the neck were excluded (Figure 1).

### 2.3. Functional Connectivity Calculation

The EEG was first band-pass filtered after which phase estimation was performed using the Hilbert transform. The Hilbert transform was implemented using a 50% overlapping sliding window approach with 250 ms long windows. It should be noted that this short window length might approach a critical length for reliable phase estimation, especially for the δ frequency band. However, using longer windows up to 4 s had virtually no influence on the DFC coefficients (results not shown). Next, the continuous phase signals were divided into 0.25 s long non-overlapping epochs which resulted in about *N* = 3,600 epochs per EEG recording. For each epoch, functional connectivity between pairs of EEG channels was calculated using the phase-lag-index (PLI, Stam et al., 2007). PLI is based on the asymmetry of the distribution of phase differences between two signals, and is calculated according to:

(1) $$\begin{array}{r} PLI=\frac{1}{T}|\overset{T}{\underset{t}{\sum }}\text{sign}\left[\sin\left(\Delta \Phi \left(t\right)\right)\right]| \end{array}$$

*T* indexes the number of samples per epoch, and ΔΦ(*t*) represents the phase difference between two signals, for sample t in radians. This resulted in a symmetric connectivity matrix of size 170 by 170 per epoch per frequency band. A PLI value of 0 indicates no functional connectivity, whereas a value of 1 indicates maximal functional connectivity. Next, the between-electrode FC matrices are reduced to between-region FC matrices of size 22 by 22. Each element, or edge, represents the average FC strength between two scalp regions for a given time interval. Based on the lateralization of each scalp region, a distinction can be made between within-hemisphere edges (LL or RR) and between-hemisphere edges (LR or RL).

### 2.4. Dynamic Functional Connectivity Calculation

Because the FC matrices are symmetric, we first transformed each 22 by 22 matrix into a 231 long vector by extracting the upper triangle. Then, for each EEG recording and each frequency band, a 231 by N matrix of time-varying FC between all pairs of scalp regions is obtained, where N indicates the number of epochs. Next, DFC is calculated by simply calculating Pearson's correlation coefficients between all pairs of time-varying FC. In this way, a symmetric “dynamic FC (DFC)” matrix of size 231 by 231 is obtained for each EEG recording and each frequency band. Each element in the DFC matrix represents the correlation strength between two time-varying FC values, referred to as “DFC coefficient.” Since the DFC matrices are symmetric, each matrix is converted to a 26565 long vector by extracting the upper triangle.

### 2.5. DFC Coefficients Types

**Figure 2:**
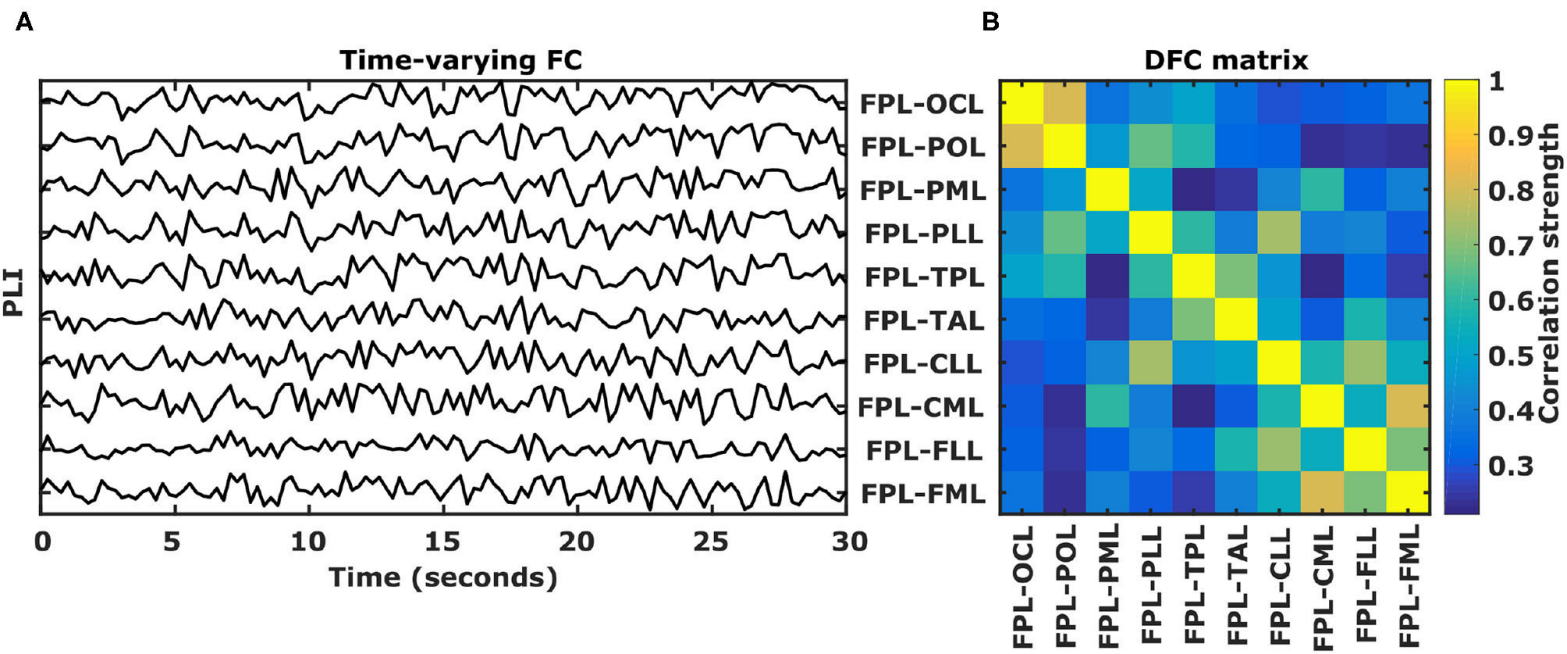
DFC calculation. **(A)** Example of 10 functional connectivity (PLI) time-series calculated for 30 s of EEG using 250 ms long epochs in the θ frequency band. PLI values range between 0 and 1 and for visualization purposes PLI time-series are stacked on top of each other. **(B)** Corresponding 10 by 10 symmetric correlation matrix of the 10 functional connectivity time-series. Each position indicates the Pearson's correlation coefficient between two PLI time-series, called a dynamic functional connectivity (DFC) coefficient. L, left hemisphere; FP, fronto-polar; FM, frontal-mid; FL, frontal lateral; CM, central mid; CL, central lateral; TA, temporal anterior; TP, temporal posterior; PL, parietal lateral; PM, parietal mid; PO, parietal occipital; OC, occipital.

To allow a more detailed analysis of identification results we used 2 ways of defining categories of DFC coefficients. One based on the number of scalp regions and another based on the lateralization of the functional connections. Since every DFC coefficient is defined as the Pearson's correlation coefficient between two functional connections, by definition each DFC coefficient involves either 3 or 4 scalp regions. In case of 22 scalp regions shown in Figure 2, this amounts to 4620 3-region and 21945 4-region DFC coefficients. Furthermore, we defined 4 categories of DFC coefficients based on which hemisphere each of the 3 or 4 scalp regions is located: 3025 unilateral within-hemispheric (LL-LL or RR-RR), 2970 unilateral between-hemispheric (LL-RR), 13310 bilateral-unilateral (LR-RR or LR-LL), and 7260 bilateral-bilateral (LR-LR).

### 2.6. Identification Analysis

In our first identification analysis we used the same approach as Finn *et al*. (Finn et al., 2015). A database was created that consisted of all the subjects' baseline DFC vectors, *D*_*set*1_ = [*X*_*i*_, *i* = 1, …, 105], where *X*_*i*_ is the DFC vector from subject *i*. Similarly, a database *D*_set2_[*Y*_*i*_, *i* = 1, …, 105], consisting of all the subjects' follow-up DFC vectors was created. In case of subject identification based on all five frequency bands, *X*_*i*_ and *Y*_*i*_ are constructed by concatenating the five 26565 long DFC vectors into a single 132825 long vector. For single-frequency band identification *X*_*i*_ and *Y*_*i*_ can either be based on the same frequency band (within-frequency band) or on different frequency bands (between-frequency band). To predict a subjects' identity in one database based on the other database, Pearson correlation between pairs of DFC vectors was defined as similarity measures and was calculated between all pairs of DFC vectors from the two databases. This resulted in a 14 by 144 similarity matrix where each element represents the similarity between a DFC vector from the baseline database and a DFC vector from the follow-up database. Subsequently, each DFC vector from one database was assigned the identity of the DFC vector from the other database that was maximally similar. Identification accuracy was defined as the percentage of subjects for which his or her identity was correctly identified. Following the approach of Finn et al. (2015), we also performed non-parametric permutation testing to assess the statistical significance of identification performance. In each iteration, identification as described above was performed whereby the identity of subjects in one database was permuted. This permutation procedure was repeated 10,000 times.

### 2.7. Gender Classification Analysis

In our gender classification analysis we used baseline data from all 144 healthy subjects. We first investigated the magnitude and extend of DFC profile differences between males and females by assessing how many DFC coefficients were statistically different (*t*-test) using a significance level of *P* < 3.76 × 10^−7^ (*P* < 0.05 Bonferroni corrected for 132,825 comparisons). Considering the heterogeneity of our data set with respect to age, male and female subsets were matched for age by randomly excluding 20 female. Presented results are obtained by averaging 500 Monte Carlo repetitions. To determine with which accuracy a subjects' gender can be predicted based on his or her DFC profile, we applied feature selection and classification using LASSO (least absolute shrinkage and selection operator) (Tibshirani, 1996). Again, presented results are obtained by averaging 500 Monte Carlo cross validation repetitions. In each repetition, 80% of the subjects are assigned to the training data set and the remaining 20% are assigned to the test data set. Furthermore, male and female subsets of the training data sets where matched for age.

### 2.8. Common DFC Blueprint Analysis

To analyze DFC profile characteristics that are shared among individuals we constructed an average gender-matched baseline DFC profile from all 144 healthy subjects. Considering the heterogeneity of our data set with respect to gender and age, an average DFC profile was created 500 times by randomly selected subset of subjects, matched for age and gender. Subsequently the 500 averaged DFC profiles where in turn averaged to obtain a population averaged $\bar{\text{DFC}}$ profile.

### 2.9. Influence of Epoch Length on DFC Information Content

To analyze the influence of epoch length used for PLI calculations on the reliability of DFC coefficients, we repeated the identification and gender classification analysis using epoch lengths ranging from 250 ms up to 32 s. Additionally, we used the EEG recordings from those healthy subjects included in the identification analysis to show that with increasing epoch lengths the test-retest error also increases. The test-retest error for a given DFC coefficient was defined as the absolute difference between its values from both recordings. Subsequently, for each DFC coefficient the errors for all subjects were averaged; the distributions of all 26,565 DFC coefficients are shown in **Figure 5**. To illustrate the effect of EEG recording duration, we simulated shorter durations by simply using only the first *N* minutes of each recording for DFC calculation. N was varied between 1 and 10 min.

## 3. Results

In the first part of this study we used data of 105 healthy subjects to show that intra-individual differences in DFC profiles are substantial and can reliably be observed, for example, they can act as an identifiable “fingerprint.” For each subject, a high-density resting-state EEG was obtained twice over a period ranging from 6 to 15 months. Detailed subject information can be found in the online material. For each subject, 12 or 15 min of eyes-closed resting state EEG data were recorded using a 256-electrode EEG recording device (Electrical Geodesics). Signals from a subset of 170 electrodes were used; midline electrodes and those located in the face and around the neck were excluded. By bandpass filtering the EEG recordings, a DFC profile was calculated for each of 5 commonly used frequency bands (δ: 1–4 Hz, θ: 4–8 Hz, α_1_: 8–10 Hz, α_2_: 10–10 Hz, and β: 13–30 Hz). This was done because distinct frequency bands exhibit characteristic changes in response to sensory, motor, and cognitive events (Engel et al., 2001; Varela et al., 2001; Buzsáki and Draguhn, 2004). It is therefore expected that DFC profiles calculated in different frequency bands will differ. Next, from the bandpass-filtered EEG, the instantaneous phases were estimated using the Hilbert transform with a 50% overlapping sliding window. Then, for 250 ms long, non-overlapping epochs, Functional Connectivity (FC) between all possible pairs of the 170 EEG channels was assessed using the phase-lag-index (PLI, Stam et al., 2007). For an EEG recording consisting of N epochs, this resulted in a series of N symmetric FC matrices of size 170 × 170. Because the localization of EEG sources is in the order of centimeters (Cohen, 2017), we reduced the 170 × 170 inter-electrode FC matrices to 22 × 22 inter-region FC matrices. Each element, or edge, represents the time-varying FC strength between two scalp regions. Thus, given 22 scalp regions, there are 231 unique edges. Finally, Pearson's correlation coefficients were calculated for all edge-pairs in order to obtain a 231 × 231 symmetric dynamic FC matrix where each element represents the correlation strength between two edge-pairs, referred to as a “DFC coefficient.” A more detailed description of the DFC calculation is provided in the section 2. DFC matrices were calculated for each EEG and for each frequency band such that each subject has 5 matrices reflecting his or her DFC profile during a session. Identification was performed using the same approach as Finn and colleagues (Finn et al., 2015). In brief, 2 datasets were constructed such that each subject had a single DFC profile in each set. Identification implied that each DFC profile in one set (“set1”) was assigned the identity of the DFC profile in the second set (“set2”) that was maximally similar. Similarity was defined as the Pearson's correlation coefficient between the two vectorized DFC profiles. Once all identities had been predicted, the overall identification rate was calculated as the percentage of scans for which the predicted identity matched the true identity. This process was then repeated with the roles of set1 and set2 reversed. Subsequently, identification performance was averaged over these two attempts.

### 3.1. Identification of Individual Subjects

#### 3.1.1. All Frequency Bands

First, identification was performed using all DFC profiles obtained from the 5 frequency bands. The Identification rate was 89/105 (85%) and 85/105 (81%) based on attempt 1 and 2, respectively. The same permutation test as used by Finn and colleagues was performed which, across 10,000 iterations, yielded a maximal identification rate of 6/105 or less than 5%. Therefore, obtaining 84 correct identifications was associated with *p* < < 0.0001.

### 3.2. Single Frequency Bands

**Figure 3:**
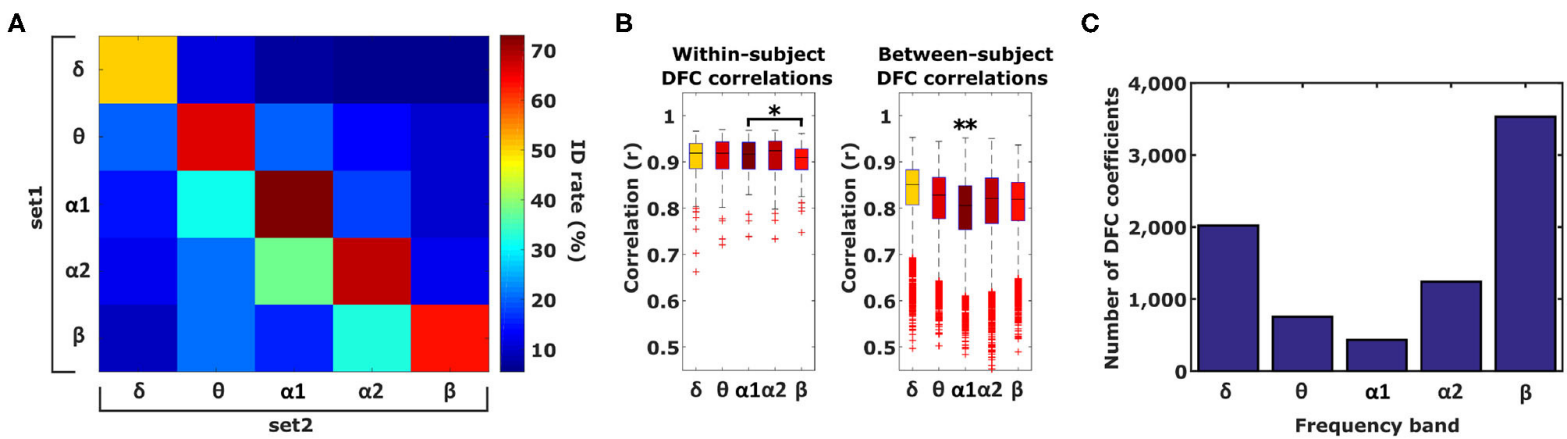
Identification and Gender classification performance. **(A)** Identification performance across different frequency band combinations, diagonal elements indicate performance of within-frequency identification and off-diagonal elements indicate between-frequency identification. **(B)** Within-subject correlation in α_1_ is only significantly higher compared to β. Between-subject correlation in *alpha*_1_ is significantly lower compared to the other 4 frequency bands (one-tailed paired *t*-test, *p < 0.01, **p ≪ 0.0001). Boxplots represent median (stripe), 25th and 75th percentiles (box), 1.5 times the interquartile range (whiskers), and outliers (crosses). Box colors correspond to the ID rate scale of **(A)**. **(C)** Number of DFC coefficients per frequency band that are statistically significant different between males and females based on a significance level of α < 3.76 × 10^−7^.

To test the hypothesis that different information, with respect to individual subject discriminability, is contained in different frequency bands, we subsequently tested identification accuracy based on DFC profiles from single frequency bands (Figure 3A). In case different information is contained in each frequency band, identification performance is expected to be lower when using single frequency bands instead of when using all 5. Furthermore, performance is expected to be reduced even more when different frequency bands are used for each set (see section 2). For within-frequency-band identification, performance indeed dropped significantly (74%, paired *t*-test α_1_ vs. all frequency bands, *t*_209_ = −3.37, *P* < 0.001). Furthermore, α_1_ identification was significantly better compared to δ, θ, and β (paired *t*-test α_1_- δ *p* = 2.5 ×10^−9^, α_1_-θ *p* = 0.028, α_1_-α_2_ *p* = 0.078, α_1_- β *p* = 0.0017). Furthermore, cross-frequency-band identification performance was significantly lower compared to within-frequency-band identification (*t*-test within- vs. cross-frequency-band identification, *t*_13_ = 8.5, *P* < 10^−5^). To asses whether better α_1_ performance can be attributed to higher within-subject correlation and/or lower between-subject correlation, we compared within- and between-subject correlations between frequency bands (Figure 3B). Compared to α_1_, within-subject correlation was only significantly lower for β (paired *t*-test, *p* < 0.01) whereas between-subject correlation was significantly higher for all 4 frequency bands (paired *t*-test, *p* ≪ 0.0001). Superior identification performance for α_1_ can therefore be attributed primarily to a relatively low between-subject similarity compared to other frequency bands.

#### 3.2.1. Gender Differences

In the previous analysis we found that an individual's DFC profile is unique, and can as such act as an identifying fingerprint. In the following analysis we investigated whether the DFC profile of a patient contains other relevant information. An important but often neglected topic in neuroscience is sex-related influences on brain function (Cahill, 2006). Recent functional and anatomical connectivity studies reported both morphometric (Gong et al., 2009; Chekroud et al., 2016), as well as functional connectivity (Tomasi and Volkow, 2012), differences between the male and the female brain. Our analysis evaluated sex-related differences, and whether DFC profiles can be used to predict an individual's gender. We used the baseline EEGs from the same 105 subjects as in the previous analysis and an additional 8 subjects for whom only a single EEG was available.

As a first step we used a *t*-test to assess how profound the sex differences of DFC profiles are. Considering the heterogeneity of our data set with respect to age, we performed 500 Monte-Carlo simulations such that in each test the male and female subset was matched according to age. On the basis of a significance level of α < 3.76 × 10^−7^ (0.05 Bonferroni-corrected for 132,825 comparisons), we found that the number of DFC coefficients that were significantly different between male and female ranged between 3,531 (13%) and 433 (1.6%) for the β and α_1_ frequency band respectively (Figure 3C). Interestingly, for each frequency band, virtually all of these DFC coefficients were higher for females. The magnitude of gender differences (male - female) ranged from −0.21 to +0.12.

**Figure 4:**
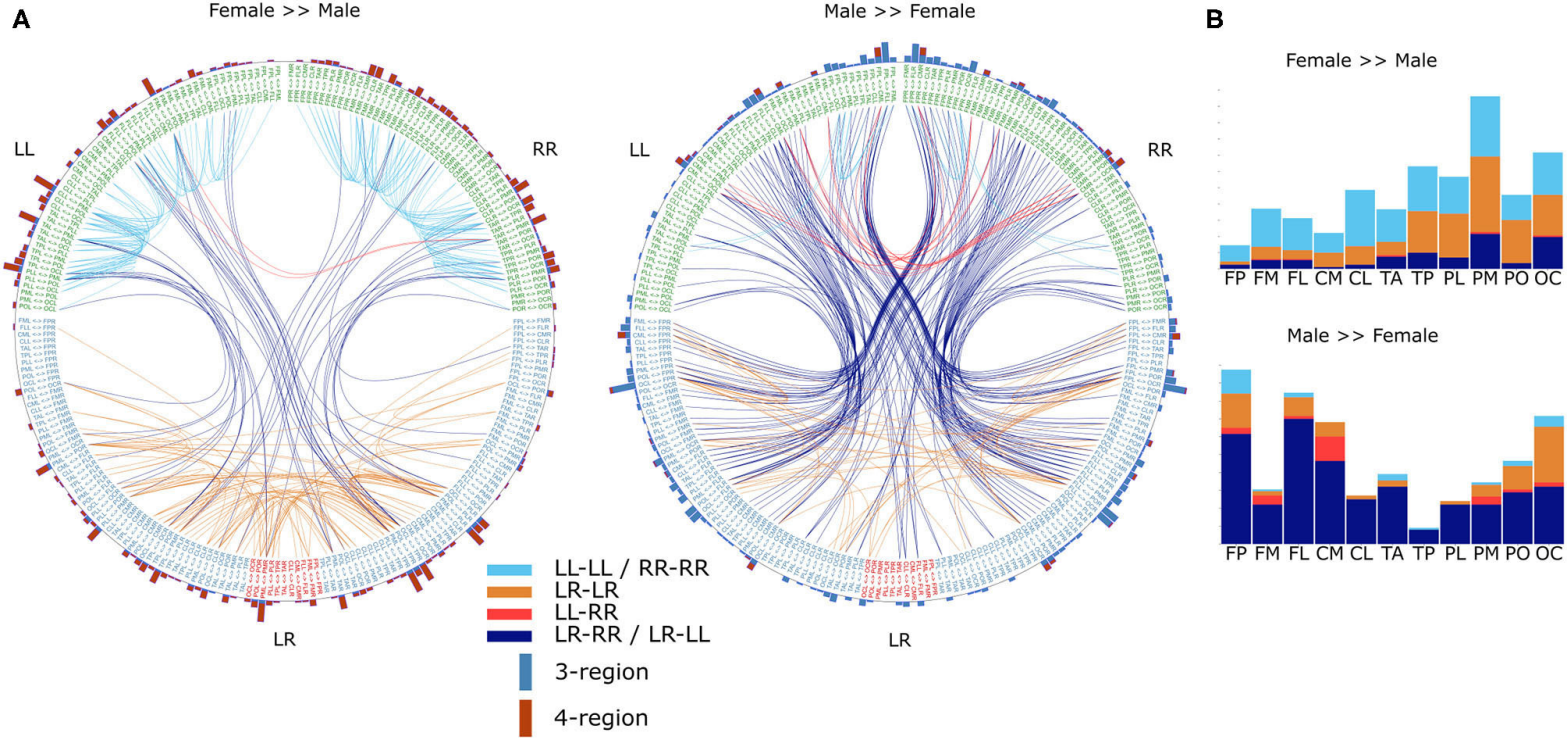
Gender differences in the α frequency band. **(A)** DFC coefficients typically stronger in female brains (left) or male brains (right). For visualization, both sets represent the top 99 percentile of DFC coefficients for which the gender difference is largest. Text on the circle represent all 231 functional connections and we made a distinction between unilateral (LL and RR) and bilateral (LR) connections. Except for connections between cross-hemisphere homologs (LR), connections are ordered such that the left and right half are mirror images. Each line represents a DFC coefficient and its color distinguishes between the 4 lateralization based categories. Additionally, on the outer circle we used a bar for each connection to summarize in how many DFC coefficients it was involved. Furthermore, each bar is divided in a blue and a red part indicating the number of 3-region and 4-region DFC coefficients, respectively. **(B)** In the barplots the same data is plotted as the incidence of scalp regions in the set of DFC coefficients, averaged over both hemispheres. L, left hemisphere; R, right hemisphere; FP, fronto-polar; FM, frontal-mid; FL, frontal lateral; CM, central mid; CL, central lateral; TA, temporal anterior; TP, temporal posterior; PL, parietal lateral; PM, parietal mid; PO, parietal occipital; OC, occipital.

The differences in the number of statistically significantly different DFC coefficients per frequency band indicate that gender differences are not the same for each frequency band. However, it is possible that the frequency bands only differ from each other with respect to gender differences in the magnitude of these differences. Therefore, we tested the hypotheses that gender differences are frequency band specific. To this end, for each frequency band f, we calculated an average male and average female DFC profile and subsequently subtracted one from the other in order to obtain a “gender difference DFC profile” (ΔDFC_f_ ). To test the hypothesis that gender differences are symmetric with respect to both hemispheres, we correlated ΔDFC with its mirrored version (see section 2). Correlation coefficients ranged from 0.9 (α_1_) to 0.94 (δ) which indicates that gender differences are indeed symmetric. We next determined for which DFC coefficient categories, and for which involved brain regions, the gender differences were most prominent. We restricted our analysis to those ΔDFC_δ_ coefficients for which the magnitude of gender difference were in the bottom 1 and top 99 percentile (Figure 4).

**Figure 5:**
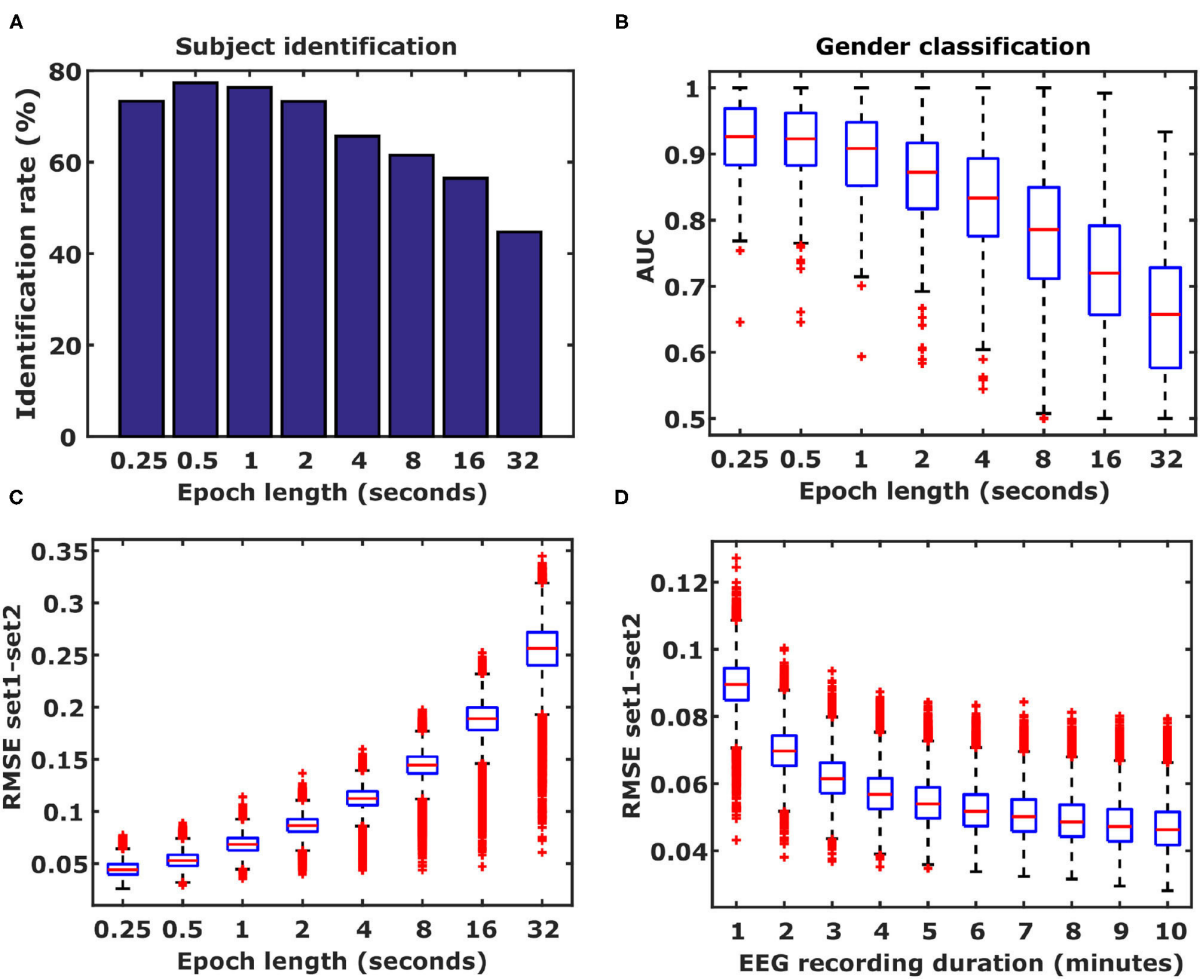
Influence of epoch length and frequency band. **(A)** Identification rates based on different epoch lengths. Epoch lengths ranged from 250 ms to 32 s. **(B)** Shorter epochs improve gender classification performance. Results from 500 Monte Carlo cross-validation repetitions are shown. Boxplots represent median (stripe), 25 th and 75th percentiles (box), 1.5 times the interquartile range (whiskers), and outliers (crosses). **(C)** Epoch length is inversely related to DFC coefficient estimation accuracy. **(D)** EEG duration is also inversely related to the duration of the EEG. Boxplots represent the distributions of differences in DFC coefficients between Rest1 and Rest2, averaged over 105 subjects for the α_1_ frequency band (26565 DFC coefficients).

To allow a more detailed analysis of the identification results, we used 2 ways of defining categories of DFC coefficients: one based on the number of regions involved in a DFC coefficient, and the other based on the lateralization of the regions involved. Another distinction can be made based on the number of regions a DFC coefficient is based on, which is either 3 or 4 regions. Lateralization is categorized according to which hemisphere each of the 3 or 4 brain regions is located: unilateral within-hemispheric (LL-LL or RR-RR), unilateral between-hemispheric (LL-RR), bilateral-unilateral (LR-RR or LR-LL), and bilateral-bilateral (LR-LR).

In case of the DFC coefficients, which were typically much stronger for females, we found that the majority were either unilateral within-hemispheric (LL-LL or RR-RR) or bilateral-bilateral (LR-LR) 4-region DFC coefficients. These DFC coefficients mainly involved parietal and occipital regions. On the other hand, DFC coefficients that were typically stronger for males were primarily bilateral-unilateral (LR-RR or LR-LL) 3-region DFC coefficients and involved primarily frontal as well as central mid, and occipital regions.

#### 3.2.2. Gender Classification

Based on the sheer number and magnitude of gender differences, it is reasonable to expect that an individual's gender can be determined based on his or her DFC profile. In the next section, we evaluated if this is indeed possible. Since not all DFC coefficients are gender-dependent, we use LASSO (least absolute shrinkage and selection operator) to evaluate if a small subset of DFC coefficients is also sufficient for gender classification. We used Monte-Carlo cross-validation where in each iteration LASSO was applied to 80% of the data in order to fit a regression model between DFC coefficients and gender indicated by 0 (male) or 1 (female). This regression model was then used to predict the gender of the left-out 20%. Prediction performance was assessed as the area under the ROC curve (AUC) of the 500 Monte-Carlo repetitions. On average, 30 (range: 12–103) DFC coefficients were selected to fit the regression model. Median classification performance was AUC = 0.93 (range: 0.6–1). In total, 3152 DFC coefficient were selected by Lasso in at least 1 of 500 repetitions. As could be expected based on the results described in the previous section, DFC coefficients from the δ (27%) and β (23%) frequency bands were chosen more frequently than the other 3 frequency bands (16% each).

#### 3.2.3. A Shared DFC Blueprint Among Individuals and Hemispheres

**Figure 6:**
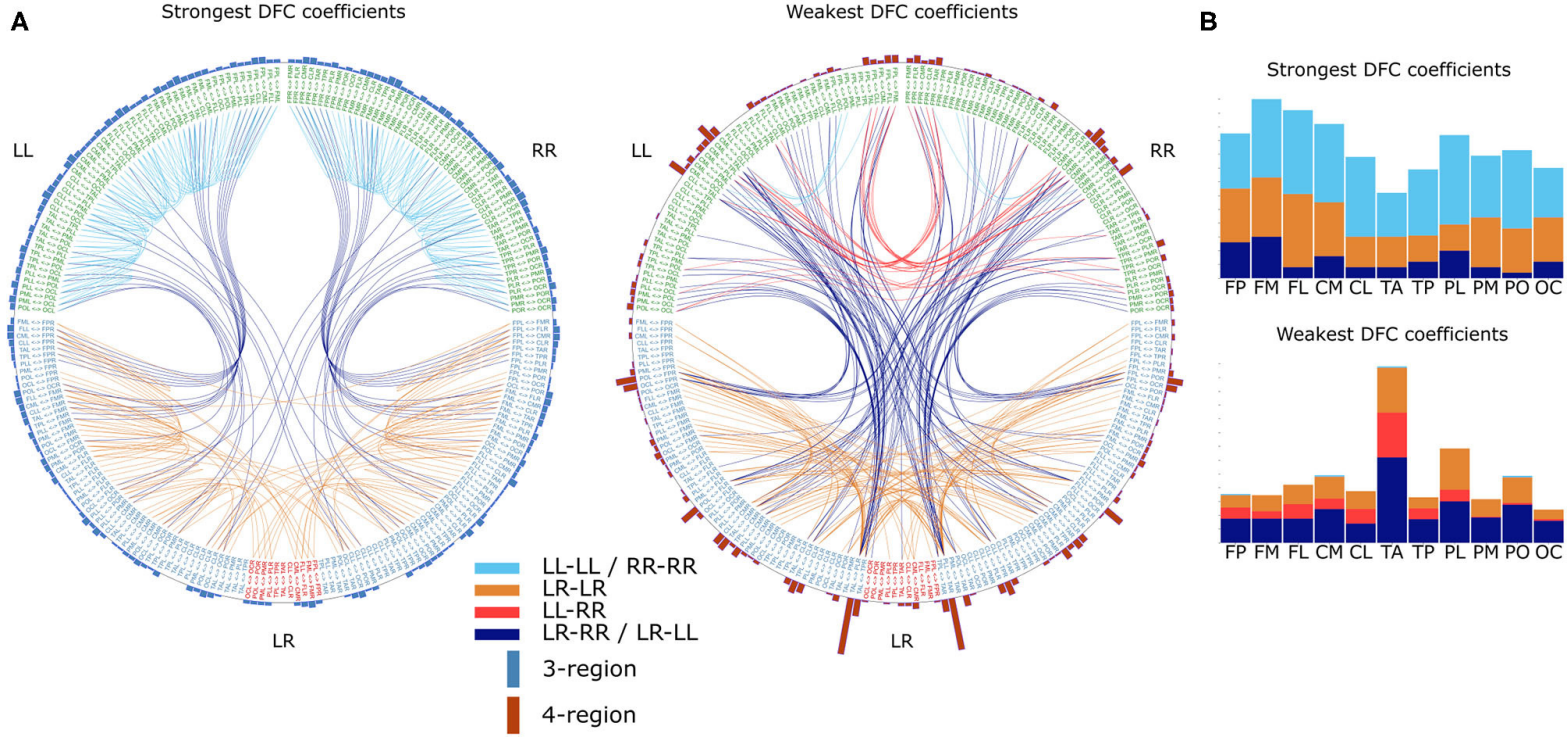
Common DFC profile characteristics in the α_1_ frequency band. **(A)** Strongest DFC coefficients (left) and weakest DFC coefficients (right) in the group averaged DFC profile. For visualization DFC coefficients in the top 99 and bottom 1 percentile are shown. Text on the circle represent all 231 functional connections and we made a distinction between unilateral (LL and RR) and bilateral (LR) connections. Except for connections between cross-hemisphere homologs (LR), connections are ordered such that the left and right half are mirror images. Each line represents a DFC coefficient and its color distinguishes between the 4 lateralization based categories. Additionally, on the outer circle we used a bar for each connection to summarize in how many DFC coefficients it was involved. Furthermore, each bar is divided in a blue and a red part indicating the number of 3-region and 4-region DFC coefficients, respectively. **(B)** In the barplots the same data is plotted as the incidence of scalp regions in the set of DFC coefficients, averaged over both hemispheres. L, left hemisphere; R, right hemisphere; FP, fronto-polar; FM, frontal-mid; FL, frontal lateral; CM, central mid; CL, central lateral; TA, temporal anterior; TP, temporal posterior; PL, parietal lateral; PM, parietal mid; PO, parietal occipital; OC, occipital. (c) Differences between the several DFC coefficient categories based on the average DFC profile of 7 all healthy individuals. Boxplots represent median (stripe), 25th and 75th percentiles (box), 1.5 times the interquartile range (whiskers), and outliers (crosses). Box colors and legend indicate DFC coefficient category.

We further investigated the common properties of DFC organization. To this end we collapsed healthy subjects' DFC matrices into a single average DFC matrix ($\bar{\text{DFC}}$). Naturally, a $\bar{\text{DFC}}$ was created for each frequency band separately. Similarly to our gender differences analysis, we here also calculated Pearson's correlation coefficients between $\bar{\text{DFC}}$ profiles of the different frequency bands, as well as between $\bar{\text{DFC}}$ and their mirrored versions. Correlation coefficients between frequency bands ranged between 0.90 (δ vs. β) and 0.99 (α_1_ vs. α_2_). Furthermore, the further apart two frequency bands were, the lower the correlation between $\bar{\text{DFC}}$ profiles. This indicates that there is a common blueprint shared among frequency bands on top of which frequency-band-specific characteristics are superposed. Conversely, correlation coefficients between $\bar{\text{DFC}}$ profiles and their mirrored version are above 0.995 for each frequency band, which indicates that both hemispheres share the same blueprint. To study this common blueprint in more detail we first evaluated $\bar{\text{DFC}}$ in terms of the different DFC coefficient categories. Considering the strong similarities between $\bar{\text{DFC}}$ from the different frequency bands, we restrict the following analysis to the α_1_ frequency band. We found that 3-region $\bar{\text{DFC}}$ coefficients are generally stronger compared to the 4-region $\bar{\text{DFC}}$ coefficients (Figure 6, *t* 26563 = 87.8, *P* ≪ 0.001). Similarly, unilateral within-hemispheric (LL-LL and RR-RR) $\bar{\text{DFC}}$ are stronger compared to the other 3 categories (Figure 6, *t* 26563 = 46, *P* ≪ 0.001). Next, we determined which $\bar{\text{DFC}}$ coefficients were in the top 99 and bottom percentile. As expected we found that unilateral within-hemispheric (LL-LL and RR-RR) as well as 3-region $\bar{\text{DFC}}$ coefficients were overly represented in the 99 percentile, and virtually absent in the 1 percentile (Figure 6). Conversely, the opposite was found for unilateral between-hemispheric and 4-region $\bar{\text{DFC}}$ coefficients (Figure 6). When analyzing the 1 and 99 percentile subsets in more detail, we found that functional connections involving frontal and parietal regions appeared most frequently in the subset of strongest $\bar{\text{DFC}}$ coefficients (Figure 6). On the other hand, in the subset of weakest $\bar{\text{DFC}}$ coefficients, functional connections involving, and especially between, temporal-anterior and parietal-lateral regions appeared most frequently.

#### 3.2.4. Influence of Epoch Length on DFC Information Content

**Figure 7:**
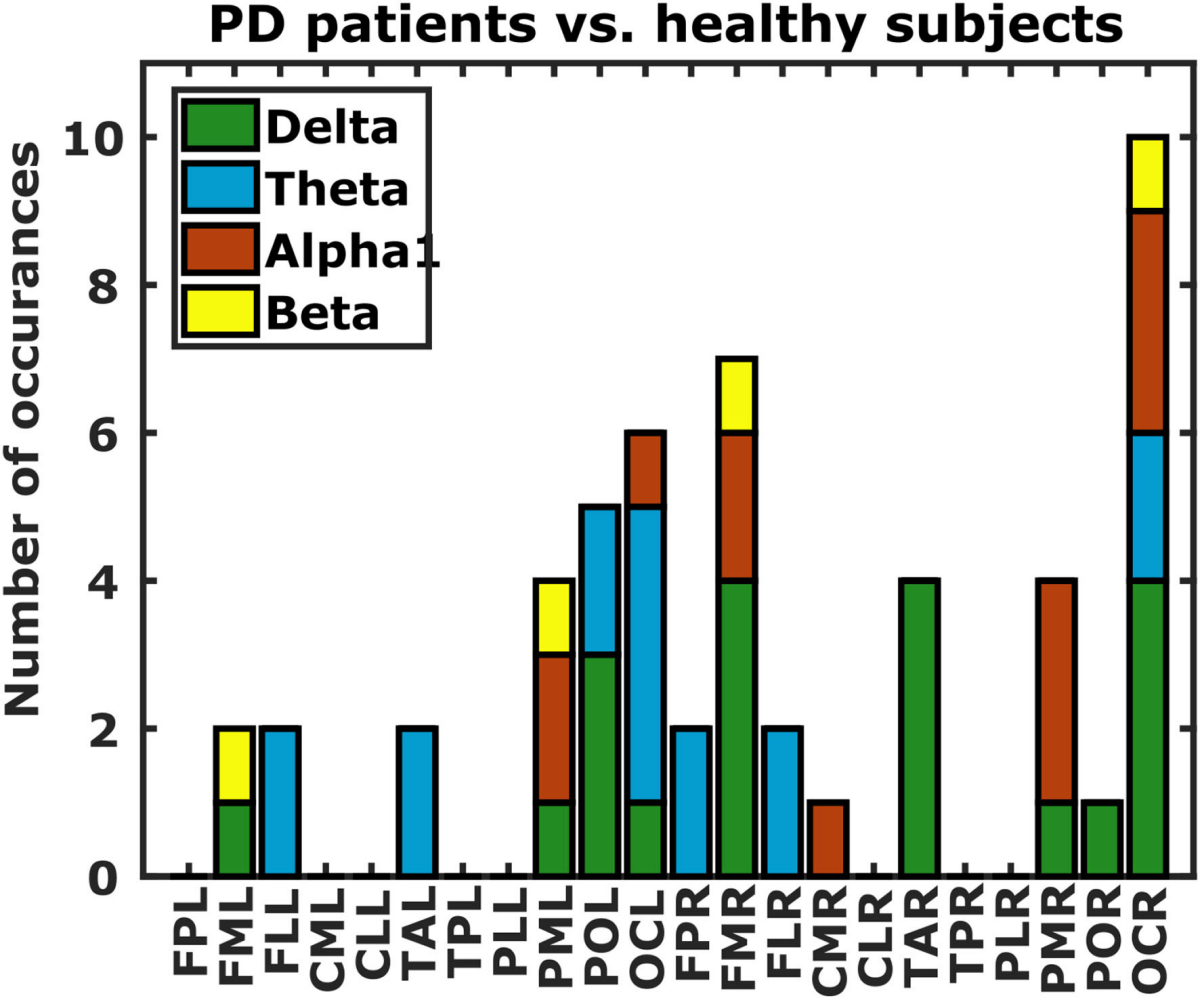
Differences in DFC profiles from PD patients and healthy subjects. Each bar indicates the number of occurrences of each of the 22 scalp regions in the set of DFC coefficients that are statistically different between PD patients and healthy subjects. Bar colors correspond to frequency bands. L, left hemisphere; R, right hemisphere; FP, fronto-polar; FM, frontal-mid; FL, frontal lateral; CM, central mid; CL, central lateral; TA, temporal anterior; TP, temporal posterior; PL, parietal lateral; PM, parietal mid; PO, parietal occipital; OC, occipital.

For very long time-windows, functional connectivity patterns closely resemble structural connectivity patterns (Foster et al., 2016). However, when functional connectivity is calculated for shorter time windows the dynamics of functional connectivity is revealed which enables analyses such as the one presented in this study. A question arises about what the effect of increasing epoch length is on the amount of information contained in the DFC coefficients. To this end, we repeated the subject identification and gender classification analyses using 7 additional epoch lengths to calculate functional connectivity: 500 ms, 1, 2, 4, 8, 16, and 32 s. Increasing the epoch length resulted in lower identification rates (Figure 7) as well as lower gender classification performance (Figure 7). This means that with decreasing epoch lengths the DFC coefficients can be estimated with higher accuracy, as expressed by the smaller differences (RMSE) in DFC coefficient values between Rest1 and Rest2 (Figure 7). Another factor that is expected to play a role in accuracy of which the DFC coefficients can be identified is the duration of the EEG recording. To test the hypotheses that shorter EEGs also result in reduced accuracy, we calculated DFC profiles with 250 ms long epochs, using truncated EEGs ranging from 1 to 10 min. We observed that shorter EEGs, and hence less data, indeed result in decreased accuracy (Figure 7).

### 3.3. External Validation: Parkinson's Disease Patients

As a stronger proof of generalizability we applied our subject identification and gender classification models on a completely independent validation set consisting of resting state EEGs from 81 patients diagnosed with Parkinson's disease (PD). Of these 81 patients, 58 had 2 EEGs recorded over a period of 4 weeks. Identification based on all 5 frequency bands resulted in an identification rate of 79%, which is in accordance with the 82% that was achieved using the 105 HC subjects. Additionally, an identification rate of 80% was achieved when identification was based on the joint set of 163 healthy subjects and PD patients.

We next applied the gender classification models from the analysis described in the “Identification and gender classification using all frequency bands” section to the 81 PD patients as well. The models trained on the data from healthy subjects could also successfully discriminate between males and females: AUC = 0.89±0.02.

Finally we investigated if DFC could also distinguish between PD patients and healthy subjects. Considering the sex-related differences in DFC coefficients, and the fact that we have almost twice as many male as female PD patients, we restricted our analysis to those DFC coefficients that were not significantly different in our gender analysis (*p* > 0.05). This yielded a total of 41616 DFC coefficients for which we assessed differences between PD patients and healthy subjects using a *t*-test. *P*-values below 1.2 × 10^−6^ (0.05 Bonferroni-corrected for 41,616 comparisons) were considered statistically significant. We also excluded a subset of healthy subjects from this analysis because they were much younger than the PD patients. Only 13 DFC coefficients survived Bonferroni correction (5, 4, 3, and 1 from the δ, θ, 1, and β frequency band respectively). All of these DFC coefficients were higher for healthy subjects, and all except 1 involved either a left or right occipital region (Figure 7).

## 4. Discussion

In practice PLI and other functional connectivity measures are typically estimated by averaging over trials and/or time(Aydore et al., 2013). Here we propose an alternative method of functional connectivity analysis. We demonstrate that correlation coefficients between pairs of functional connectivity time-series contain meaningful information about the functional organization of the brain. First, we demonstrated that an individual's DFC profile is unique and reliably observable which makes it possible to use it to identify an individual from a large group of subjects. Second, we also demonstrate that an individual's DFC profile is highly gender-specific, which makes it possible to determine a subject's gender solely based on his or her DFC profile. Third, we show that there is a general blueprint for DFC organization that is not only shared among individuals, but also between left and right hemispheres. These observations are in accordance with our current understanding of functional brain organization, namely, that there is an intrinsic standard architecture for functional brain organization on top of which task- (Cole et al., 2014) and individual-specific characteristics (Finn et al., 2015) are superposed. Furthermore, this architecture is also shared between left and right hemispheres which agrees with observations that similar bilateral functional networks develop independently (Tyszka et al., 2011). Fourth, our results also generalize to a population of PD patients, and furthermore indicate that there are specific changes in DFC profiles from PD patients. This observation in particular is promising because it suggests that DFC can be used to study neurodegenerative diseases. Last, we showed that DFC coefficients dependent on the frequency band used to calculate PLI values. A large body of research links specific patterns of oscillations to perceptual, cognitive, motor, and emotional processes (Cohen, 2017). It is therefore reasonable to expect that DFC profiles calculated based on different frequency bands are associated with different neuronal processes.

### 4.1. DFC Coefficient Interpretation

A DFC coefficient is defined as the correlation strength between two PLI time-series. First, it is important to recognize that PLI calculated over a period of several hundreds of milliseconds cannot reflect an intrinsic property of the brain's functional organization. This is because the magnitude of epoch length and the typical duration of phase-locking are the same (Varela et al., 2001). Consequently, during most epochs the instantaneous phases of two signals only cross once, twice, or not at all. Assuming that only 1 phase-crossing occurs, the resulting PLI value only depends on when during this epoch the phase-crossing took place. Naturally, in such a scenario a single PLI value is determined by chance. However, if two PLI time-series are positively correlated, it means that the phase crossing in both sets of signals repeatedly occur around the same time. Hence, a DFC coefficient can best be understood as a measure of the timing relationship of the phase crossings between sets of EEG signals. Contrary to single PLI values calculated over short epochs, it is reasonable to assume the timing relationship between phase-crossing does reflect an intrinsic property of the brain.

Our observation that the majority of DFC coefficients are stronger in females fits to other studies demonstrating stronger anatomical (Gong et al., 2009) as well as functional connectivity (Tomasi and Volkow, 2012). However, we also found a subset of DFC coefficients which were stronger in males. This is in line with observations that some functional connections are stronger in females while others are stronger in males (Biswal et al., 2010). If our hypothesis of higher DFC coefficients reflecting shorter time-intervals between phase-crossings is correct, differences in brain size might also partly explain differences in DFC coefficients between the sexes (Hänggi et al., 2014). Why gender differences are most profound in the δ (1–4 Hz) and β (13–30 Hz) frequency bands, and least profound in the α_1_ (8–10 Hz) frequency band, might be explained by the opposite pattern observed in our subject identification analysis, where between-subject DFC correlations were lowest in the α_1_ (8–10 Hz) frequency band. However, caution is warranted when trying to find a physiological interpretation for a measure of statistical dependence between EEG signals. A method such as DFC might indicate which EEG features are relevant for deeper investigation. For example, it would be interesting to use an effective connectivity approach, such as direct causal modeling (Friston, 2011), to better understand the mechanism of brain connectivity.

### 4.2. Additional Considerations

The development of our DFC method is an empirical extension of existing FC methods, and therefore based on several assumptions such as the use of epochs, averaging over 22 scalp regions, PLI as functional connectivity measure, and choice of frequency bands. However, it is not straightforward that these assumptions are valid, let alone optimal, when studying the dynamics of FC. For example, one could argue that for 250 ms-long epochs, it does not make sense to use PLI as a FC measure. Nevertheless, we showed that our DFC method provide meaningful information about an individual. Therefore, our DFC method can best be seen as the starting point of developing theoretically sounder methods to study the dynamics of phase interactions.

We deliberately used minimal pre-processing, in the form of band-pass filtering, and also used the complete resting state EEG recording. A common approach in quantitative EEG analysis is to let one or more EEG experts select parts of an EEG recording which are then used for subsequent analysis, instead of using the complete recording. Although this might prevent the results being negatively influenced by various artifacts, it also obscures the process of how the results are obtained. Furthermore, it enables cherry-picking of EEG data such that better results are obtained. However, it goes without saying that careful selection of high-quality EEG data should remain part of any quantitative EEG analysis, especially when used to draw inference on individual subjects.

## 5. Conclusion

These results underline the potential of EEG-based DFC as a tool to investigate brain functioning on both a population and individual level. This could in turn lead to the discovery of new “neuromarkers” for cognitive behavior, psychiatric conditions, and cognitive decline in neurodegenerative diseases. Furthermore these results underline the validity of phase-based functional connectivity methods such as PLI, be it as an intermediate step for a more advanced quantification of functional brain organization.

## Data Availability Statement

The raw data supporting the conclusions of this article will be made available by the authors, without undue reservation.

## Ethics Statement

The studies involving human participants were reviewed and approved by Ethikkommission Beider Basel, Nordwestschweiz. The patients/participants provided their written informed consent to participate in this study.

## Author Contributions

GB, UG, PF, and VR conceptualized the study. GB, MC, FH, and AM collected and processed the data. GB designed and performed the analyses, wrote the data visualization tools, and wrote the manuscript, with comments from all authors. GB and FH wrote the analysis tools. PF and VR provided guidance with data interpretation. The study was performed under the supervision of PF and VR.

## Conflict of Interest

The authors declare that the research was conducted in the absence of any commercial or financial relationships that could be construed as a potential conflict of interest.

## Publisher's Note

All claims expressed in this article are solely those of the authors and do not necessarily represent those of their affiliated organizations, or those of the publisher, the editors and the reviewers. Any product that may be evaluated in this article, or claim that may be made by its manufacturer, is not guaranteed or endorsed by the publisher.

## References

[B1] Allen E. Damaraju E. Eichele T. Wu L. Calhoun V. D. (2018). EEG signatures of dynamic functional network connectivity states. Brain Topogr. 31, 101–116. 10.1007/s10548-017-0546-2 28229308 PMC5568463

[B2] Allen E. A. Damaraju E. Plis S. M. Erhardt E. B. Eichele T. Calhoun V. D. (2014). Tracking whole-brain connectivity dynamics in the resting state. Cereb. Cortex 24, 663–676. 10.1093/cercor/bhs352 23146964 PMC3920766

[B3] Aydore S. Pantazis D. Leahy R. M. (2013). A note on the phase locking value and its properties. Neuroimage 74, 231–244. 10.1016/j.neuroimage.2013.02.008 23435210 PMC3674231

[B4] Barttfeld P. Uhrig L. Sitt J. D. Sigman M. Jarraya B. Dehaene S. (2015). Signature of consciousness in the dynamics of resting-state brain activity. Proc. Natl. Acad. Sci. U.S.A. 112, 887–892. 10.1073/pnas.1418031112 25561541 PMC4311826

[B5] Bassett D. S. Sporns O. (2017). Network neuroscience. Netw. Neurosci. 20:353. 10.1038/nn.4502 PMC5485642 28230844

[B6] Biswal B. B. Mennes M. Zuo X.-N. Gohel S. Kelly C. Smith S. M.. (2010). Toward discovery science of human brain function. Proc. Natl. Acad. Sci. U.S.A. 107, 4734–4739. 10.1073/pnas.0911855107 20176931 PMC2842060

[B7] Buzsáki G. Draguhn A. (2004). Neuronal oscillations in cortical networks. Science 304, 1926–1929. 10.1126/science.1099745 15218136

[B8] Cahill L. (2006). Why sex matters for neuroscience. Nat. Rev. Neurosci. 7, 477–484. 10.1038/nrn1909 16688123

[B9] Calhoun V. D. Miller R. Pearlson G. Adalı T. (2014). The chronnectome: time-varying connectivity networks as the next frontier in fMRI data discovery. Neuron 84, 262–274. 10.1016/j.neuron.2014.10.015 25374354 PMC4372723

[B10] Chekroud A. M. Ward E. J. Rosenberg M. D. Holmes A. J. (2016). Patterns in the human brain mosaic discriminate males from females. Proc. Natl. Acad. Sci. U.S.A. 113, E1968. 10.1073/pnas.1523888113 26984491 PMC4833246

[B11] Chen J.-L. Ros T. Gruzelier J. H. (2013). Dynamic changes of ICA-derived EEG functional connectivity in the resting state. Hum. Brain Mapp. 34, 852–868. 10.1002/hbm.21475 22344782 PMC6870341

[B12] Cohen M. X. (2017). Where does EEG come from and what does it mean? Trends Neurosci. 40, 208–218. 10.1016/j.tins.2017.02.004 28314445

[B13] Cole M. W. Bassett D. S. Power J. D. Braver T. S. Petersen S. E. (2014). Intrinsic and task-evoked network architectures of the human brain. Neuron 83, 238–251. 10.1016/j.neuron.2014.05.014 24991964 PMC4082806

[B14] Damaraju E. Allen E. A. Belger A. Ford J. M. McEwen S. Mathalon D.. (2014). Dynamic functional connectivity analysis reveals transient states of dysconnectivity in schizophrenia. Neuroimage Clin. 5, 298–308. 10.1016/j.nicl.2014.07.003 25161896 PMC4141977

[B15] de Pasquale F. Della Penna S. Snyder A. Z. Marzetti L. Pizzella V. Romani G. L.. (2012). A cortical core for dynamic integration of functional networks in the resting human brain. Neuron 74, 753–764. 10.1016/j.neuron.2012.03.031 22632732 PMC3361697

[B16] Dubois J. Adolphs R. (2016). Building a science of individual differences from fMRI. Trends Cogn. Sci. 20, 425–443. 10.1016/j.tics.2016.03.014 27138646 PMC4886721

[B17] Engel A. K. Fries P. Singer W. (2001). Dynamic predictions: oscillations and synchrony in top-down processing. Nat. Rev. Neurosci. 2, 704–716. 10.1038/35094565 11584308

[B18] Finn E. S. Shen X. Scheinost D. Rosenberg M. D. Huang J. Chun M. M.. (2015). Functional connectome fingerprinting: identifying individuals using patterns of brain connectivity. Nat. Neurosci. 18:1664. 10.1038/nn.4135 26457551 PMC5008686

[B19] Foster B. L. He B. J. Honey C. J. Jerbi K. Maier A. Saalmann Y. B. (2016). Spontaneous neural dynamics and multi-scale network organization. Front. Syst. Neurosci. 10:7. 10.3389/fnsys.2016.00007 26903823 PMC4746329

[B20] Friston K. J. (2011). Functional and effective connectivity: a review. Brain Connect. 1, 13–36. 10.1089/brain.2011.0008 22432952

[B21] Gong G. Rosa-Neto P. Carbonell F. Chen Z. J. He Y. Evans A. C. (2009). Age-and gender-related differences in the cortical anatomical network. J. Neurosci. 29, 15684–15693. 10.1523/JNEUROSCI.2308-09.2009 20016083 PMC2831804

[B22] Gonzalez-Castillo J. Hoy C. W. Handwerker D. A. Robinson M. E. Buchanan L. C. Saad Z. S.. (2015). Tracking ongoing cognition in individuals using brief, whole-brain functional connectivity patterns. Proc. Natl. Acad. Sci. U.S.A. 112, 8762–8767. 10.1073/pnas.1501242112 26124112 PMC4507216

[B23] Hänggi J. Fövenyi L. Liem F. Meyer M. Jäncke L. (2014). The hypothesis of neuronal interconnectivity as a function of brain size–a general organization principle of the human connectome. Front. Hum Neurosci. 8:915. 10.3389/fnhum.2014.00915 25426059 PMC4227509

[B24] Hatz F. Meyer A. Zimmermann R. Gschwandtner U. Fuhr P. (2017). Apathy in patients with Parkinson's disease correlates with alteration of left fronto-polar electroencephalographic connectivity. Front. Aging Neurosci. 9:262. 10.3389/fnagi.2017.00262 28860987 PMC5559507

[B25] Honey C. J. Kötter R. Breakspear M. Sporns O. (2007). Network structure of cerebral cortex shapes functional connectivity on multiple time scales. Proc. Natl. Acad. Sci. U.S.A. 104, 10240–10245. 10.1073/pnas.0701519104 17548818 PMC1891224

[B26] Hutchison R. M. Morton J. B. (2015). Tracking the brain's functional coupling dynamics over development. J. Neurosci. 35, 6849–6859. 10.1523/JNEUROSCI.4638-14.2015 25926460 PMC6605187

[B27] Hutchison R. M. Womelsdorf T. Allen E. A. Bandettini P. A. Calhoun V. D. Corbetta M.. (2013). Dynamic functional connectivity: promise, issues, and interpretations. Neuroimage 80, 360–378. 10.1016/j.neuroimage.2013.05.079 23707587 PMC3807588

[B28] Liu Q. Farahibozorg S. Porcaro C. Wenderoth N. Mantini D. (2017). Detecting large-scale networks in the human brain using high-density electroencephalography. Hum. Brain Mapp. 38, 4631–4643. 10.1002/hbm.23688 28631281 PMC6867042

[B29] Näpflin M. Wildi M. Sarnthein J. (2007). Test-retest reliability of resting EEG spectra validates a statistical signature of persons. Clin. Neurophysiol. 118, 2519–2524. 10.1016/j.clinph.2007.07.022 17892969

[B30] Shiino A. Chen Y. W. Tanigaki K. Yamada A. Vigers P. Watanabe T.. (2017). Sex-related difference in human white matter volumes studied: Inspection of the corpus callosum and other white matter by VBM. Sci. Rep. 7, 1–7. 10.1038/srep39818 28045130 PMC5206615

[B31] Snyder A. C. Morais M. J. Willis C. M. Smith M. A. (2015). Global network influences on local functional connectivity. Nat. Neurosci. 18:736. 10.1038/nn.3979 25799040 PMC4641678

[B32] Stam C. J. Nolte G. Daffertshofer A. (2007). Phase lag index: assessment of functional connectivity from multi channel EEG and MEG with diminished bias from common sources. Hum. Brain Mapp. 28, 1178–1193. 10.1002/hbm.20346 17266107 PMC6871367

[B33] Tagliazucchi E. Von Wegner F. Morzelewski A. Brodbeck V. Laufs H. (2012). Dynamic bold functional connectivity in humans and its electrophysiological correlates. Front. Hum. Neurosci. 6:339. 10.3389/fnhum.2012.00339 23293596 PMC3531919

[B34] Tibshirani R. (1996). Regression shrinkage and selection via the lasso. J. R. Stat. Soc. Ser. B Methodol. 58, 267–288. 10.1111/j.2517-6161.1996.tb02080.x

[B35] Tomasi D. Volkow N. D. (2012). Gender differences in brain functional connectivity density. Hum. Brain Mapp. 33, 849–860. 10.1002/hbm.21252 21425398 PMC3250567

[B36] Tyszka J. M. Kennedy D. P. Adolphs R. Paul L. K. (2011). Intact bilateral resting-state networks in the absence of the corpus callosum. J. Neurosci. 31, 15154–15162. 10.1523/JNEUROSCI.1453-11.2011 22016549 PMC3221732

[B37] Van Diessen E. Numan T. Van Dellen E. Van Der Kooi A. Boersma M. Hofman D.. (2015). Opportunities and methodological challenges in EEG and MEG resting state functional brain network research. Clin. Neurophysiol. 126, 1468–1481. 10.1016/j.clinph.2014.11.018 25511636

[B38] Varela F. Lachaux J.-P. Rodriguez E. Martinerie J. (2001). The brainweb: phase synchronization and large-scale integration. Nat. Rev. Neurosci. 2, 229–239. 10.1038/35067550 11283746

[B39] Yu Q. Wu L. Bridwell D. A. Erhardt E. B. Du Y. He H.. (2016). Building an EEG-fMRI multi-modal brain graph: a concurrent EEG-fMRI study. Front. Hum. Neurosci. 10:476. 10.3389/fnhum.2016.00476 27733821 PMC5039193

